# Out-Door Safety

**Published:** 1879-12

**Authors:** 


### OUT-DOOR SAFETY.

THE fear of the weather has sent multitudes to the grave, who otherwise might have lived in health many years longer. The fierce north wind and the furious snow-storm kill comparatively few, while hot winter rooms and crisping summer suns have countless hecatombs of human victims to attest their power. Except in localities where malignant miasms prevail, and that only in warm weather, out-door life is the healthiest and happiest, from the tropics to the poles.

The general fact speaks for itself, that persons who are out of doors most, take cold least. In some parts of our country, near one half of the adult deaths are from diseases of the air passages. These ailments arise from taking cold in some way or another; and surely the reader will take some interest in a subject, which, by at least one chance out of four, his own life may be lost.

All colds arise from one of two causes.

1st. By getting cool too quick after exercise, either as to the whole body, or any part of it.

2d. By being chilled, and remaining so for a long time, from want of exercise.

To avoid colds from the former, we have only to go to a fire the moment the exercises cease in the winter. If in summer, repair at once to a closed room, and there remain with the same clothing on, until cooled off.

To avoid colds from the latter cause, and these engender the most speedily fatal diseases, such as pleurisies, croup, and inflammation of the lungs, called pneumonia , we have only to compel ourselves to walk with sufficient vigor to keep off a feeling of chilliness. Attention to a precept contained in less than a dozen words, would add twenty years to the average of civilized life.

*Keep away chilliness by exercise; cool off slowly.* Then you will never take cold, in door or out!

---

To see those we love a source of happiness to others, is a pure joy.

